# Training patients for self‐administration of a new subcutaneous C1‐inhibitor concentrate for hereditary angioedema

**DOI:** 10.1002/nop2.194

**Published:** 2018-08-28

**Authors:** Elyse Murphy, Christine Donahue, Laurel Omert, Stephanie Persons, Thomas J. Tyma, Joseph Chiao, William Lumry

**Affiliations:** ^1^ CSL Behring King of Prussia Pennsylvania; ^2^ Allergy, Asthma, Immunology Medical Care Portland Oregon; ^3^ Asthma, Allergy & Immunology Associates, Ltd. Scottsdale Arizona; ^4^ AARA Research Center Dallas Texas

**Keywords:** C1‐INH(SC), C1‐inhibitor, HAEGARDA, hereditary angioedema, prophylaxis, self‐administration, subcutaneous

## Abstract

**Aims:**

The aim of this study was to provide recommendations for training patients with hereditary angioedema, based on nursing clinical trial experience, to self‐administer subcutaneous C1‐INH (C1‐INH[SC]) used as routine prophylaxis.

**Background:**

A volume‐reduced, subcutaneous C1‐INH concentrate (C1‐INH(SC); HAEGARDA®; CSL Behring) was recently FDA‐approved for the routine prevention of hereditary angioedema attacks. Nurses will play an important role in patient training.

**Design:**

Review of a phase 3, randomized, placebo‐controlled, double‐blind, crossover trial of C1‐INH(SC) (COMPACT) and summary of recommendations for training patients based on nurses’ “hands‐on experience.”

**Methods:**

A panel of nurses with clinical trial experience provided recommendations for patient training.

**Results:**

Practical suggestions and guidelines were compiled regarding patient selection, product reconstitution and administration and patient follow‐up. Successful patient self‐administration of C1‐INH(SC) can be greatly facilitated by qualified nursing intervention. The information provided in this paper will be useful to nurses anywhere who have an opportunity to interact with patients dealing with hereditary angioedema.

**What does this paper contribute to the wider global clinical community?**

- Hereditary angioedema (HAE) is a rare but highly morbid and potentially life‐threatening genetic condition for which new and better treatment options continue to emerge.
- Subcutaneous C1‐inhibitor (C1‐INH) is the newest option for routine prevention of HAE attacks approved by the US FDA in June 2017 and expected to be available in other global markets as early as 2018

## 1 INTRODUCTION

Hereditary angioedema (HAE) is a rare but potentially life‐threatening genetic condition caused by either a quantitative deficiency or qualitative dysfunction of the C1‐inhibitor (C1‐INH) protein that serves as an important regulator in the contact system. The absence of C1‐INH regulation activity results in increased bradykinin production which leads to vascular permeability and leakage of oedema fluid (angioedema) (Zuraw & Christiansen, 2016). This type of angioedema is nonhistaminergic, which becomes important when choosing therapy, as typical treatments for histamine‐mediated oedema (e.g., antihistamines) are not effective in HAE. HAE type 1 (low levels of C1‐INH) accounts for about 85% of cases of HAE and is associated with a quantitative deficiency of C1‐INH (Bernstein, 2013; Bork, 2014). Patients with HAE type II (approximately 15% of cases) have normal or elevated levels of C1‐INH but abnormal C1‐INH functionality. Clinically, types 1 and 2 are indistinguishable. A third type more recently identified, HAE with normal C1‐INH, is much less common and has in some cases been associated with factor XII mutations (Zuraw et al., 2012).

**Figure 1:**
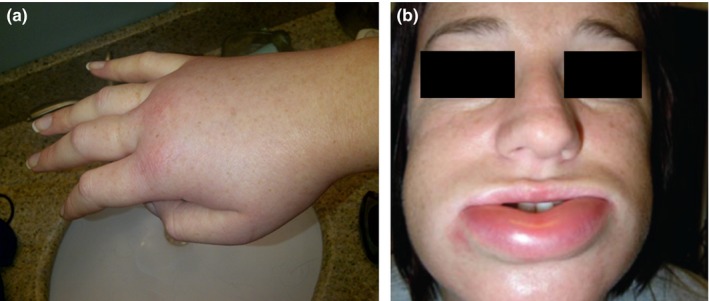
Peripheral (a) and facial (b) swelling associated with hereditary angioedema attacks

Hereditary angioedema is characterized by recurrent episodes of nonpruritic subcutaneous or submucosal swelling that most commonly affect the extremities, the face, the trunk and abdomen, and the genitals (Bork, Meng, Staubach & Hardt, 2006). The frequency and severity of HAE attacks are generally unpredictable but may be precipitated by triggering events such as physical trauma (e.g., medical and dental procedures), emotional stress and hormonal changes. Untreated attacks can last for hours to days and can be acutely painful, disfiguring, debilitating and life‐threatening (Banerji, 2013). Figure 1 contains photos of patients with peripheral and facial HAE attacks. Laryngeal attacks have the potential to cause permanent disability or death and can occur without warning or history of such attacks (Bork, Hardt, & Witzke, 2012). Abdominal attacks with swelling of the intestinal wall are experienced by a majority of patients with HAE; pain, vomiting and diarrhoea associated with these types of attacks can be a significant source of distress and disability and can be misdiagnosed as an acute surgical abdomen process (Bork, Meng, et al., 2006; Bork, Staubach, Eckardt & Hardt, 2006). Considering the chronic, lifelong nature of HAE and the disruptiveness and unpredictability of HAE attacks, it is not surprising that most patients with this disease report a significant impact on their health‐related quality of life (HRQOL) (Banerji et al., 2015). The psychological distress caused by HAE may also potentially contribute to anxiety and depression, as suggested by elevated rates of these disorders in the HAE population (Aygören‐Pürsün et al., 2014; Banerji, 2013; Caballero et al., 2014, 2013; Fouche, Saunders, & Craig, 2014; Lumry et al., 2010).

### 1.1 Background

The goals of HAE management are to reduce morbidity, prevent mortality and improve quality of life to the greatest extent possible. Specific treatment strategies include on‐demand treatment of attacks, short‐term prophylaxis for anticipated triggers (e.g., surgery, a stressful life event) and/or routine long‐term prophylaxis to prevent angioedema attacks (Cicardi et al., 2014, 2012; Craig et al., 2012; Zuraw et al., 2013). It is recommended that all patients with HAE have access to on‐demand therapy (Cicardi et al., 2012; Craig et al., 2012; Lang et al., 2012; Zuraw et al., 2013). HAE‐specific medications that are available for the treatment of attacks when they occur include two IV products: C1‐INH concentrate (Berinert®; CSL Behring, Marburg, Germany) and recombinant human C1‐INH concentrate (Ruconest®; Pharming, Inc., Leiden, the Netherlands) and two subcutaneous (SC) products: the bradykinin B2 receptor antagonist icatibant (Firazyr®, Shire, Lexington, MA, USA) and a kallikrein inhibitor, ecallantide (Kalbitor®, Shire, Lexington, MA, USA).

Routine long‐term prevention is considered for patients in whom on‐demand acute therapy is inadequate to minimize the personal burden related to HAE (Cicardi et al., 2012; Craig et al., 2012; Lang et al., 2012; Zuraw et al., 2013). The ideal therapy for this purpose would eliminate attack risk with a high degree of safety and impose minimal burden of its own. Historically, oral attenuated androgens (e.g., danazol, stanozolol) were the primary options for routine prophylaxis in patients with HAE. However, their use is associated with undesirable side effects such as weight gain, acne, headaches in most patients and additionally, virilization and menstrual irregularities in female patients, as well as more serious risks of hepatic adenomas and hepatocellular carcinoma with long‐term use (Riedl, 2015; Zuraw, Davis, Castaldo, & Christiansen, 2016). In 2008, an intravenous formulation of C1‐INH concentrate ([C1‐INH(IV)]; Cinryze®; Shire ViroPharma Biologics, Inc.; Lexington, MA, USA) became available for routine prophylaxis of HAE attacks (Cinryze PI, 2015). Routine prophylaxis with intravenous (IV) C1‐INH concentrate has been shown to reduce HAE attack frequency (Bernstein et al., 2014; Zuraw et al., 2010) and improve quality of life in patients (Greeve et al., 2016; Lumry, Miller, Newcomer, Fitts, & Dayno, 2014). The most recent international HAE management guidelines from the World Allergy Organization and the European Academy of Allergy and Clinical Immunology support the use of C1‐INH as a first‐line option for prophylaxis (Maurer et al., 2018). Recommended dosing is 1,000–2,500 IU given IV every 3–4 days. Patients can be trained to self‐administer C1–INH(IV) and this practice has become widely implemented in the US (Riedl, Banerji, & Gower, 2015) and Europe (Aygören‐Pürsün et al., 2015). Yet, IV self‐administration presents particular challenges related to venous access, safety risks of thrombosis and infection from indwelling port use (which some patients prefer to have surgically implanted) and patient convenience issues (Zuraw et al., 2013). In a recent survey of C1‐INH(IV) users, more than half reported having problems finding usable veins or other administration problems (Riedl et al., 2017). Further, routine prevention with C1‐INH(IV) is not completely effective and many patients continue to experience breakthrough attacks while using it (Zuraw et al., 2010). Ongoing fear of attacks can contribute to higher levels of anxiety and depression in patients with HAE, as has been shown in patients in Europe and the US, even in patients being managed with available medications over recent years (Caballero et al., 2013; Christiansen et al., 2015; Fouche et al., 2014).

#### 1.1.1 Subcutaneous C1‐INH

A C1‐INH concentrate formulated for subcutaneous administration (C1‐INH[SC]; CSL830; HAEGARDA®; CSL Behring, Marburg, Germany) for the routine prevention of HAE attacks (Longhurst et al., 2017; Zuraw et al., 2015) was approved by the US FDA in 2017. Future approvals are anticipated in non‐US regions including Europe, Australia and Canada. The FDA‐approved dose of C1‐INH(SC) is 60 IU/kg of body weight, given twice weekly (every 3 or 4 days).

The clinical safety and efficacy of C1‐INH(SC) were confirmed in the COMPACT (Clinical Study for Optimal Management of Preventing Angioedema with Low‐Volume Subcutaneous C1‐inhibitor Replacement Therapy) study, a randomized, placebo‐controlled, dose‐ranging, double‐blind, crossover trial designed to test the hypothesis that twice‐weekly administration of C1‐INH(SC) could reduce the frequency of HAE attacks compared with placebo (Longhurst et al., 2017). The study enrolled males and females. Ninety patients ≥12 years of age with HAE type I or II who experienced at least 4 HAE attacks over 2 months prior to screening were randomized to one of four treatment sequences: self‐administered C1‐INH(SC) 40 IU/kg (*N* = 45) or 60 IU/kg (*N* = 45) twice weekly for 16 weeks, preceded or followed by twice weekly placebo for 16 weeks.

Full results of the COMPACT study have been published elsewhere (Longhurst et al., 2017). In summary, the mean number of HAE attacks per month was significantly lower during treatment with C1‐INH(SC) 60 IU/kg than during treatment with placebo (0.5 vs. 4.0 attacks; within‐patient difference, −3.5; *p* < 0.001); the median reduction in attack rate was 95% and there was a >99% reduction in the use of rescue medications to treat attacks. In addition, the average severity of HAE attacks experienced while on C1‐INH(SC) 60 IU/kg was notably less than during placebo use and no subjects (0%) had a laryngeal attack during the 16 week treatment period with 60 IU/kg C1‐INH(SC), whereas nine subjects in this group suffered 12 laryngeal attacks while on placebo. It should be kept in mind that treatment in the COMPACT trial was only 16 weeks and these findings do not guarantee that laryngeal attacks will never occur while using C1‐INH(SC) 60 IU/kg.

The percentage of patients reporting any adverse event (AE) while using C1‐INH(SC) 60 IU/kg (69.8%) was similar to the percentage of patients reporting AEs while using placebo (66.3%) (Chiao, Li, Banerji, & Jacobs, 2017). Injection site reactions, which were solicited from patients, were the most commonly documented AEs and were noted at least once in 34.9% of patients (15 of 43) while using C1‐INH(SC) 60 IU/kg and 7.8% of all C1‐INH(SC) 60 IU/kg injections (103 of 1,302 injections). A majority (85.4%) of all reported injection site reactions during use of C1‐INH(SC) 60 IU/kg were mild in severity and none were classified as severe.

Th C1‐INH(SC) formulation offers patients with HAE an effective new treatment option that gives them a proactive role in management of their disease. Self‐administered SC injections can be fit into their schedule and can be injected every 3 or 4 days, or as prescribed by their doctor, at a time and place that is most convenient for them. The dose is based on body weight, so the patient gets the dose they personally need to prevent attacks. Nurses will play an important part in training and supporting patients in self‐administration of C1‐INH(SC). The purpose of this paper is to provide recommendations for training patients to self‐administer C1‐INH(SC) based on the cumulative experience of a panel of infusion nurses who were involved in the COMPACT study, combined with official product recommendations and other relevant published information.

## 2 METHODS

A panel of infusion nurses who participated in the COMPACT clinical trial convened in June 2016 to share experiences pertaining to training patients on self‐administration of C1‐INH(SC). Systematic questions pertaining to administration techniques, patient teaching and reactions and troubleshooting and monitoring were used to extract clinical trial experience and compile recommendations for patient training based on majority consensus.

## 3 RESULTS

### 3.1 Selecting appropriate patients for self‐administration of C1‐INH(SC)

Surveys of real‐life HAE treatment patterns (Aygören‐Pürsün, Martinez‐Saguer, Rusicke, Klingebiel, & Kreuz, 2010; Jolles et al., 2014; Riedl, 2015; Riedl et al., 2016) indicate an increasing trend towards self‐administration of HAE medications. This approach allows patients greater control of their disease management, provides flexibility that may permit patients to lead more normal lives and has been associated with increased HRQOL (Boysen, Bouillet, & Aygören‐Pürsün, 2013; Bygum, Andersen, & Mikkelsen, 2009). In addition, evidence suggests that patients who receive HAE prophylaxis in the home setting may be more adherent to treatment than patients receiving therapy at physicians’ offices or infusion centres, possibly because of the added convenience and the reduced travel requirement (Gregory, Landmesser, Corrigan, & Mariano, 2014).

International guidelines recommend that all patients with HAE be considered for self‐administration if they are willing (Boysen et al., 2013; Craig et al., 2012; Longhurst et al., 2010). Appropriate patients for self‐administration of HAE medication must be motivated and willing to invest the time and effort necessary to learn self‐administration, must be mentally and physically capable of preparing and self‐injecting their treatment and should demonstrate reliability (e.g., keeping scheduled appointments) (Shapiro & Zacek, 2014). Adolescents may be considered for home administration if a responsible adult is willing to undertake training (Longhurst et al., 2010). Patients of advanced age may also be considered for self‐administration if they are willing and able to function safely and effectively, either alone or with a partner. In addition to evaluating the patient's appropriateness for self‐administration, it is often the responsibility of the nurse to visit the home to ensure that it provides a safe environment for medication storage and administration (Shapiro & Zacek, 2014).

### 3.2 Patient education and training

Although some patients may initially be intimidated by the idea of self‐administration of SC injections, the provision of appropriate education, training and counselling will allow most patients to feel comfortable with the process (Li, 2016; Shapiro & Zacek, 2014; Symons, Rossi, Magerl, & Andritschke, 2013) and there is a long history of patients with other chronic diseases (e.g., diabetes, primary immune deficiency disease) mastering self‐administered SC injections. Patient training needs to be individualized. Most patients are capable of learning self‐injection. However, the amount of time required will vary from patient to patient. If applicable, parents and/or caregivers should be trained at the same time. Patients should be taught the value of planning ahead and getting into a routine, such as setting up a regular schedule that allows sufficient time from beginning to end. The patient/caregiver must learn to master the following skills: reconstitution of C1‐INH(SC), injection site and needle/syringe preparation (including aseptic technique) and injection of C1‐INH(SC).

#### 3.2.1 Reconstitution of C1‐INH(SC)

C1‐INH(SC) is provided as a freeze‐dried powder that must be reconstituted with Sterile Water for Injection, USP (provided). C1‐INH(SC) should be stored between temperatures of 2º–30ºC (36º–86ºF), should not be frozen and should be protected from light. The final concentration of the reconstituted solution is 500 IU/ml. The following supplies are provided as part of a kit and should be assembled prior to reconstitution:

- Vial(s) of lyophilized C1‐INH(SC) concentrate, room temperature
- Vial(s) of diluent, room temperature
- Mix2Vial transfer set (any commercially available double‐ended needle and vented filter spike can be used)
- Alcohol swabs

Table 1 outlines recommended steps for the reconstitution of C1‐INH(SC). To ensure successful reconstitution using the Mix2Vial transfer set, the bottom vial should always be placed on a firm surface, not hand‐held, when attaching vials to the transfer set. It is important that the correct vial be used in each step. If the vacuum is lost, the vial contents can be mixed manually; patients should be instructed how to do this.

**Table 1:** Reconstitution of C1‐INH(SC)

Step	Directions	Illustration
1	Choose a flat surface, like a table and clean thoroughly with an alcohol swab.	
2	Wash hands thoroughly with warm soapy water.	
3	Place the C1‐INH(SC) vial, the diluent vial and the Mix2Vial on the clean, flat surface. Ensure that both vials are at room temperature.	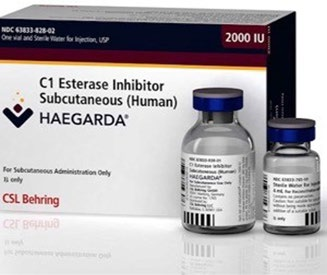
4	Remove flip caps from the diluent vial and product vial; wipe rubber stoppers with alcohol swab and allow to dry.	
5	Peel the lid from the Mix2Vial transfer device; do not remove device from package.	
6	Place the diluent vial on a flat surface and hold the vial tightly. Grip the Mix2Vial transfer set together with the clear packaging and push the plastic spike at the blue end of the Mix2Vial transfer set firmly through the centre of the stopper of the diluent vial.	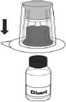
7	Then, carefully remove the clear package from the Mix2Vial transfer set without removing it or touching the exposed end.	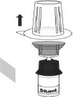
8	With the C1‐INH(SC) vial placed firmly on a flat surface, invert the diluent vial with the Mix2Vial transfer set and push the plastic spike of the transparent adapter firmly through the centre of the stopper of the product vial. The diluent will automatically transfer into the C1‐INH(SC) vial.	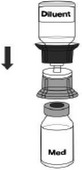
9	With the diluent and product vial still attached to the Mix2Vial, gently swirl the product to ensure that the powder is fully dissolved. Do not shake the vial. It can take up to 10 min for the product to dissolve completely.	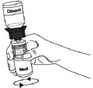
10	With one hand, grasp the C1‐INH(SC) vial and with the other hand grasp the coloured diluent side of the Mix2Vial transfer set and unscrew the set into two pieces.	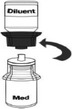
11	Draw air into an empty, sterile, silicon‐free syringe. While the product vial is upright, screw the syringe to the Mix2Vial transfer set. Inject air into the product vial.	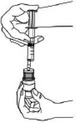
12	While keeping the syringe plunger pressed, invert the system upside down and draw the concentrate into the syringe by pulling the plunger back slowly.	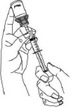
13	Disconnect the filled syringe by unscrewing it from the Mix2Vial transfer set.	
14	Visually inspect the final solution. The reconstituted solution should be colourless, clear and free from visible particles. It should not be used if particulate matter or discoloration is observed.	
15	The reconstituted solution should be used within 8 hr and stored at room temperature.	
16	The filled syringe should be attached to a hypodermic needle or subcutaneous infusion set and the plunger gently pushed to fill the needle or tubing.	
17	If the dose requires more than one vial, use a separate, unused Mix2Vial transfer set and diluent vial for each product vial.	

#### 3.2.2 Injection site and needle/syringe preparation

**Figure 2:**
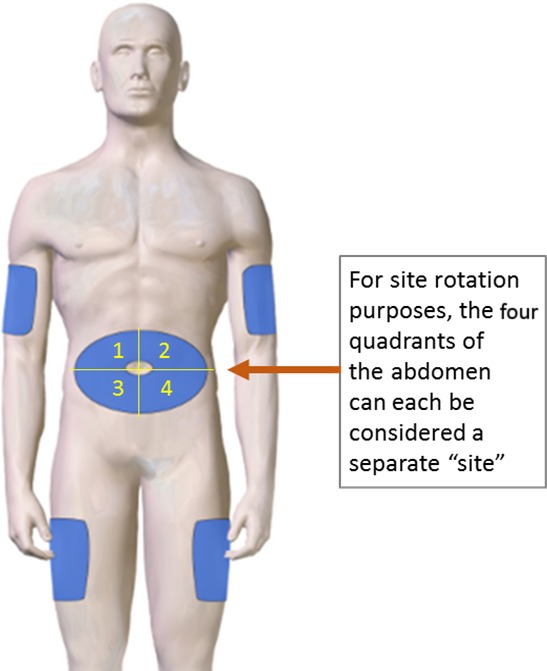
Common subcutaneous injection sites

Injection technique and choice of ancillary supplies can be tailored and adjusted to maximize comfort of injections. C1‐INH(SC) can be injected in the abdominal area or other typical SC injection sites (Figure 2). Care should be taken to avoid injecting into skin or tissue that is itchy, swollen, painful, bruised or red, as well as areas with scars or stretch marks. The injection site should be rotated from dose to dose to avoid using the same site repeatedly. It is recommended that new injection sites be at least 2 inches (5 cm) away from the previous injection site. Prior to injection, the skin at the injection site should be cleaned with an alcohol swab and allowed to dry, as residual alcohol may cause additional injection site pain/burning and/or erythema.

**Figure 3:**
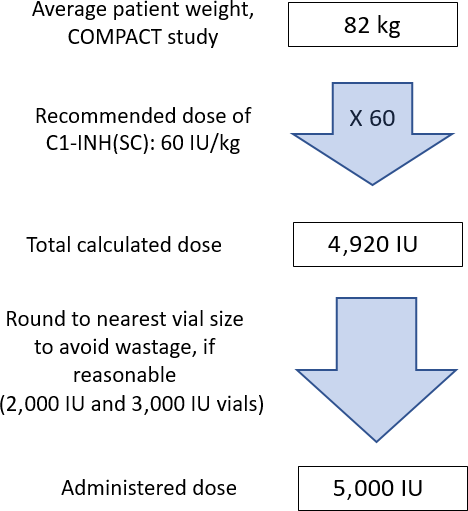
Example of C1‐INH(SC) dose calculation and vial rounding; average patient from COMPACT study

Single‐use vials of C1‐INH(SC) come in two sizes: a 2,000 IU vial with 4 ml sterile water for reconstitution and a 3,000 IU vial with 6 ml sterile water. Reconstituted vials contain C1‐INH(SC) at a concentration of 500 IU/ml. While not part of official dosing recommendations, patients in the COMPACT study were instructed to round doses up to a whole vial quantity to avoid wastage (Longhurst et al., 2017). As shown in the dose calculation example provided in Figure 3, a typical adult dose would be roughly 10 ml. During the COMPACT study (all patients), dose volumes greater than 10 ml were used at least once by 38 (42.2%) patients; only one patient used a dose volume exceeding 20 ml (21 ml).

The syringe containing the reconstituted C1‐INH(SC) solution can be attached to either a hypodermic needle or a SC infusion set. During C1‐INH(SC) clinical trials, both types of administration sets were available. Most patients in these trials preferred using a SC infusion set. Although hypodermic needles can be harder to push, some patients preferred this technique. In general, large doses (e.g., ≥10 cc) are easier to administer if split between two syringes (administered at one or two sites, depending on patient preference).

The needle length should be individualized to adequately reach the subcutaneous layer. In the COMPACT study, patients had a choice between three needle types: a SC injection needle (Hypodermic Pro‐Edge), a SC infusion set with a 9 mm needle and a SC infusion set with a 12 mm needle. Approximately one‐quarter of patients tried more than one type of needle. There were no apparent trends to suggest an overall preference for any specific needle type. Some patients felt the 12 mm subcutaneous needles were associated with fewer injection site reactions and less leakage compared with shorter needles. For thin patients, 9 mm subcutaneous needles may be adequate and preferred.

#### 3.2.3 Injection of C1‐INH(SC)

C1‐INH(SC) can be self‐administered without the use of an infusion pump. The recommended steps for injecting C1‐INH(SC) are described in Table 2. Mastery is achieved when the patient can perform all necessary steps without prompting; referral to printed materials should be allowed and encouraged if necessary. A systematic training checklist of reconstitution and injection steps can be a helpful training aid and reference. Once all steps have been mastered, patients should be able to reconstitute and administer the injection within about 15–30 min. Patients who are already accustomed to using injectable medication will likely train more quickly than those who are not, while patients who are naïve to injectable medication use may require more training. A “see one, do one, teach one” approach will ensure that the patient has achieved competency. A demo injection belly (“dummy tummy”), or subcutaneous practice pad, can be a helpful training aid, especially for patients naïve to SC injections.

**Table 2:** Injection of C1‐INH(SC)

Step	Directions	Illustration
1	Gently pinch clean skin between thumb and fingers.	
2	Remove cap from needle. If using a SC infusion set: Bend and hold wings between thumb and index finger.	
3	Whether using a SC infusion set or hypodermic syringe, the needle should be inserted under the skin at a 90° or 45° angle. The tip of the needle has to pass through the skin layer but not be so deep as to reach the muscle. Factors such as needle length and thickness of the subcutaneous layer will determine the required angle of injection.	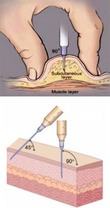
4	If using a SC infusion set, a sterile dressing can be placed over the injection site to secure the needle.	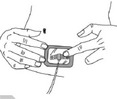
5	The plunger should be pulled back slightly. If any blood is observed in the syringe/tubing, the needle and any tubing should be discarded and replaced. While the syringe with the product can still be used, the injection should be reattempted at a new site.	
6	The syringe plunger is slowly pushed to deliver the C1‐INH(SC) dose.	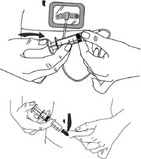
7	Rate of injection Basic rule: Push slowly enough for comfort/tolerability. An approximate guide for injection rate is about 1 ml/min. Patients can slow or increase the injection rate according to their comfort level and tolerability. If a subcutaneous “bubble” or swelling develops, injection may be too fast and/or too shallow.	
8	When infusion is finished, needle is removed and discarded appropriately per local requirements.	
9	Patients should be encouraged to record the C1‐INH(SC) lot number in a diary or treatment log book.	

Note

The information and descriptions provided here are based on clinical trial nurses’ experience; for official instructions, please refer to the C1‐INH(SC)/HAEGARDA Prescribing Information.

#### 3.2.4 What to expect from a SC injection of C1‐INH(SC)

**Figure 4:**
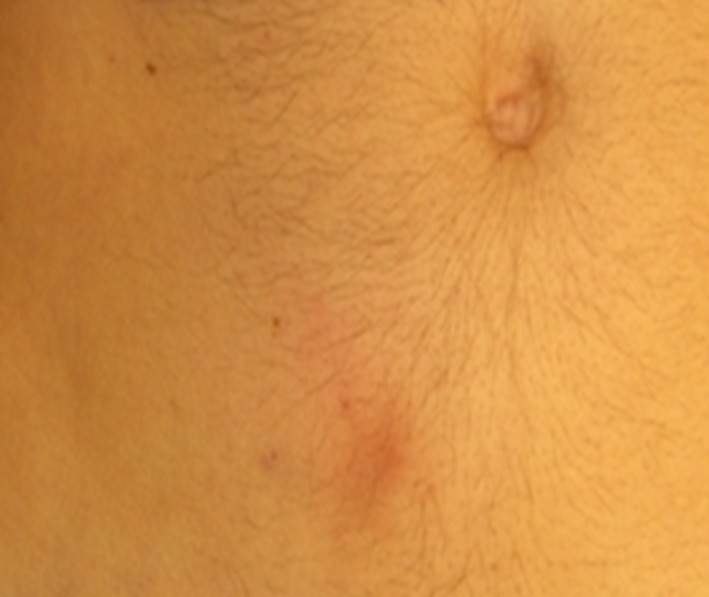
Typical mild injection site reaction following a subcutaneous injection

As with any type of injection, some degree of redness or other local effects are quite normal with the use of C1‐INH(SC). As discussed previously, injection site reactions with C1‐INH(SC) were common during clinical trials so patients should be prepared for such, yet also reassured that the vast majority of such reactions are mild. Figure 4 illustrates a typical mild injection site reaction. In addition, the majority of injection site reactions during C1‐INH(SC) 60 IU/kg use resolved quickly, within 24 hr (62.1%) or 48 hr (28.2%) and all resolved completely. While some degree of pain would be expected with a SC injection, pain at the injection site was only reported by 16% of C1‐INH(SC) 60 IU/kg users in the COMPACT study, despite all patients being asked. Patients should also understand that some local swelling at the injection site is not uncommon, especially with injected volumes greater than 5–10 ml at a single site.

### 3.3 Follow‐up care

It is critical to keep in close contact with patients during the first few months of C1‐INH(SC) use. The frequency of follow‐up will depend on the patient, their ability and skill level. Weekly contact for the first month or two may be advisable, with the nature of the contact dictated largely by patient preference (phone, email, SMS text, etc.). Patients should be encouraged to keep a diary or log book to document their injections and treatment compliance. It is also recommended that patients document and report any breakthrough HAE attacks, their treatment and unusual/lingering symptoms to ensure that all symptoms are being treated appropriately and to help assess the patient's ability to manage their condition (Boysen et al., 2013).

A variety of resources and programs for healthcare providers and patients are available from the manufacturer. These are described on the HAEGARDA product website which includes a toll‐free phone number for additional information and support access.

### 3.4 Breakthrough attack strategy

All patients should have an on‐demand treatment available in the case of a breakthrough attack. Any of the available on‐demand therapies for HAE (plasma‐derived C1‐INH concentrate, recombinant C1‐INH concentrate, the kallikrein inhibitor ecallantide and the bradykinin B2 receptor antagonist icatibant) can be used to treat breakthrough attacks in patients using C1‐INH(SC). It is important to note that C1‐INH(SC) should not be used to treat an HAE attack. Oral androgens, sometimes used for prophylaxis, are also not appropriate for on‐demand treatment. Patients should be encouraged to have a “go bag” ready with on demand HAE medication, an HAE letter (from their HAE caregiver with pertinent information for emergency caregivers who may not be familiar with HAE) and whatever else is needed for acute treatment of breakthrough attacks. The patient should be aware of the location of the closest emergency department. In rural areas, local first responders should be made familiar with the basics of HAE and what to do if called on.

## 4 CONCLUSIONS AND RELEVANCE TO CLINICAL PRACTICE

Hereditary angioedema is a burdensome, lifelong disease with significant morbidity and potential mortality. C1‐INH(SC) is a safe and effective option for prevention of HAE attacks that offers patients improved disease management. A subcutaneous C1 inhibitor may help overcome many of the challenges associated with IV administration and may provide patients with greater convenience and flexibility in managing their condition. With proper nursing training and support, patients can learn to self‐administer C1‐INH(SC). A successful transition from the IV to SC formulation can be greatly facilitated by qualified nursing intervention, not to mention patients who have never used injectable medications. The practical information provided in this paper will be useful to nurses anywhere who have an opportunity to interact with patients dealing with HAE.

## CONFLICTS OF INTEREST

E Murphy, C Donahue, L Omert, and J Chiao are employees of CSL Behring, the manufacturer of C1‐INH(SC) and financial sponsor of this manuscript. S Persons, T Tyma, and W Lumry received a stipend for participation in the C1‐INH(SC) nursing advisory board. T Tyma and S Persons report receiving travel reimbursements to attend investigator meetings for CSL Behring, Dyax, and Biocryst. W Lumry received grants and honoraria from CSL Behring, Dyax, Biocryst, and Shire.
